# A case report on the diagnosis and treatment of a glioneuronal tumor with ATRX alteration, kinase fusion, and anaplastic features

**DOI:** 10.3389/fonc.2026.1748551

**Published:** 2026-01-23

**Authors:** Siyuan He, Yao Yang, Jiezhou Zheng, Mengqi Sun

**Affiliations:** Department of The Second Clinical Medical College, Jinan University; Department of Radiation Oncology, Shenzhen People’s Hospital, Shenzhen, Guangdong, China

**Keywords:** case report, glioneuronal tumor, GTAKA, NTRK fusions, re-irradiation, temozolomide

## Abstract

Glioneuronal tumor with ATRX alteration, kinase fusion, and anaplastic features (GTAKA) is a rare pathological subtype of the central nervous system, with currently limited research available. This case report details a 37-year-old woman who developed a new intracranial tumor 22 years after initial treatment (surgical resection and adjuvant radiotherapy) for a WHO grade 2 astrocytoma. The new lesion was histopathologically diagnosed as GTAKA, a distinct glioneuronal tumor entity, following which the patient received adjuvant radiotherapy with concomitant temozolomide chemotherapy. Although this pathological type is rare, the immunohistochemical and molecular findings, combined with the therapeutic approach in this case, contribute to a deeper understanding of the disease.

## Introduction

In 2023, Bogumil et al. first described glioneuronal tumors with NTRK mutations, kinase fusions, and anaplastic features (GTAKA) as a distinct subtype of high-grade glioma. Current evidence suggests that GTAKA predominantly occurs in children or young adults, with no significant sex predilection (1). Due to the limited understanding of this entity, no standardized treatment guidelines exist. This case report details the diagnostic and therapeutic course of a patient with GTAKA, aiming to provide new clinical insights for managing this rare pathological subtype.

## Case presentation

This case report was prepared in accordance with the CARE guidelines (2). The patient initially underwent resection of a third ventricular tumor in 2003, achieving gross total removal. The postoperative pathology at that time suggested a WHO grade 2 astrocytoma (pathology report from an external hospital; specific pathological images are unavailable). The original pathological slides could not be retrieved due to the significant time elapsed. Following surgery, the patient received adjuvant radiotherapy (50 Gy in 25 fractions of 2 Gy) at Shenzhen People’s Hospital but did not undergo chemotherapy. Regular follow-up with contrast-enhanced magnetic resonance (MR) imaging of the head was conducted. The last follow-up MR scan in 2016 showed no imaging evidence of tumor recurrence or progression. However, the patient voluntarily discontinued regular follow-up thereafter.

In July 2025, the patient presented to Shenzhen Second People’s Hospital with symptoms of vomiting, lethargy, followed by syncope and seizures. A contrast-enhanced MR imaging of the head revealed findings suggestive of tumor recurrence at the site of the previously resected “astrocytoma” in the third ventricle. Susceptibility-weighted imaging (SWI) also indicated multiple micro-hemorrhages in the bilateral cerebellum, occipital lobes, and temporal lobes. Subsequently, the patient underwent a left frontal lobe and lateral ventricle glial tumor resection procedure combined with an external ventricular drain placement under general anesthesia at the same hospital. According to the patient’s provided hospitalization records, a near-total resection (NTR) of the glioma was achieved.

The postoperative pathological diagnosis from Shenzhen Second People’s Hospital was as follows:

Gross Specimen Descriptions: (Left lateral ventricular tumor) The specimen consisted of grayish-yellow to grayish-brown tissue fragments, aggregating to 2.5 cm × 1.8 cm × 1.0 cm in total size, with a partially gelatinous appearance. The cut surface was solid, soft, and grayish-white to grayish-yellow.

**Figure 1:**
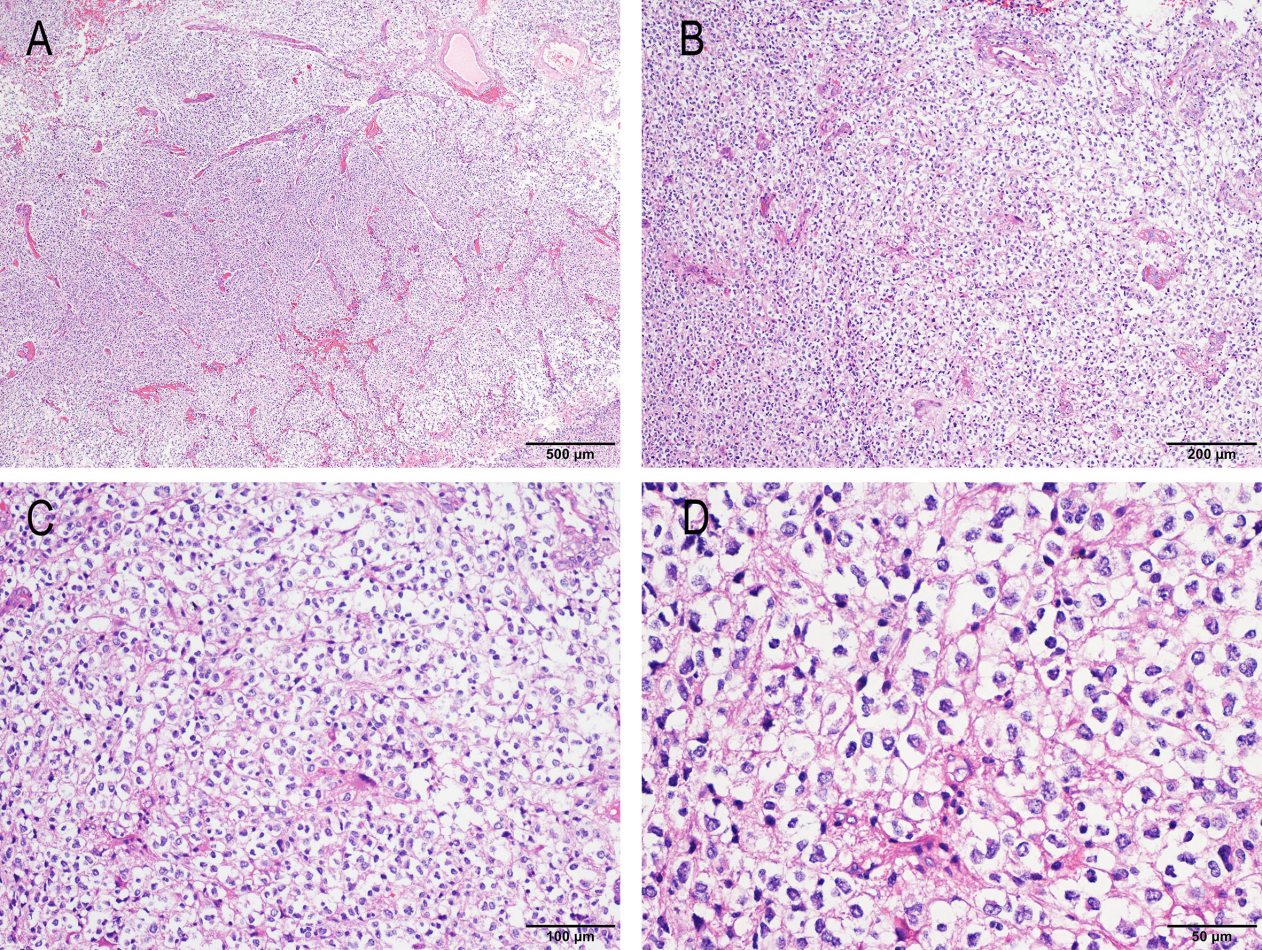
HE staining of the surgical specimen of GTAKA from the left frontal lateral ventricle. **(A)** HE staining, 40x magnification; **(B)** HE staining, 100x magnification; **(C)** HE staining, 200x magnification; **(D)** HE staining, 400x magnification.

Microscopic Description: The tumor showed proliferating cells. Some areas had a relatively clear boundary with the surrounding brain tissue. The cellular density was moderate, with most cells exhibiting an oligodendrocyte-like morphology featuring clear cytoplasm and centrally located, round nuclei. Mitotic figures were readily observed, and microvascular proliferation was present. No obvious necrosis was identified (**Figure 1**).

Integrated Diagnosis: (Left lateral ventricle) Glioneuronal Tumor with ATRX Alteration, Kinase Fusion, and Anaplastic Features (GTAKA); IDH-wildtype.

Histological Diagnosis: High-grade neuroepithelial tumor with oligodendrocyte-like histology and microvascular proliferation.

The tumor specimen was also referred to Sun Yat-sen University Cancer Center for pathological consultation. The consultation confirmed the lesions in the left lateral ventricle and left frontal lobe lateral ventricle. Immunohistochemical analysis revealed the following results: Olig-2 (positive), SOX-10 (positive), Syn (positive), H3K27Me3 (positive), NKX2.2 (partially positive), GFAP (partially positive), Ki-67 (30% positive), Calretinin (focally positive),P53 (strongly and weakly positive, heterogeneous), IDH1 (negative), ATRX (negative, indicating loss), H3K27M (negative), BRAF (negative), NeuN (negative), CD34 (negative), TIF-1 (negative), EMA (negative), D2-40 (negative). Molecular pathology results demonstrated: IDH1 gene mutation (sequencing, wildtype), IDH2 gene mutation (sequencing, wildtype), TERT promoter mutation (sequencing, wildtype), 1p/19q co-deletion (FISH, negative, no deletion detected), CDKN2A deletion (FISH, negative, no homozygous deletion of CDKN2A gene detected), and BRAF gene mutation (PCR, wildtype).

The integrated diagnosis was a glioneuronal tumor with ATRX mutation, kinase fusion, and anaplastic features (GTAKA). According to the CNS WHO grading, this case was considered equivalent to grade 3. Additional molecular information from an external laboratory included next-generation sequencing (NGS) results: an ATRX p.E1133Dfs*2 mutation (47.30%), a MYO5A-NTRK3 fusion (14.64%), a UBE2Q2-NTRK3 fusion (29.66%), MGMT promoter methylation positive (33.75%), and 1p deletion. Methylation clustering analysis yielded a result supportive of GTAKA with a score of 0.93.

**Figure 2:**
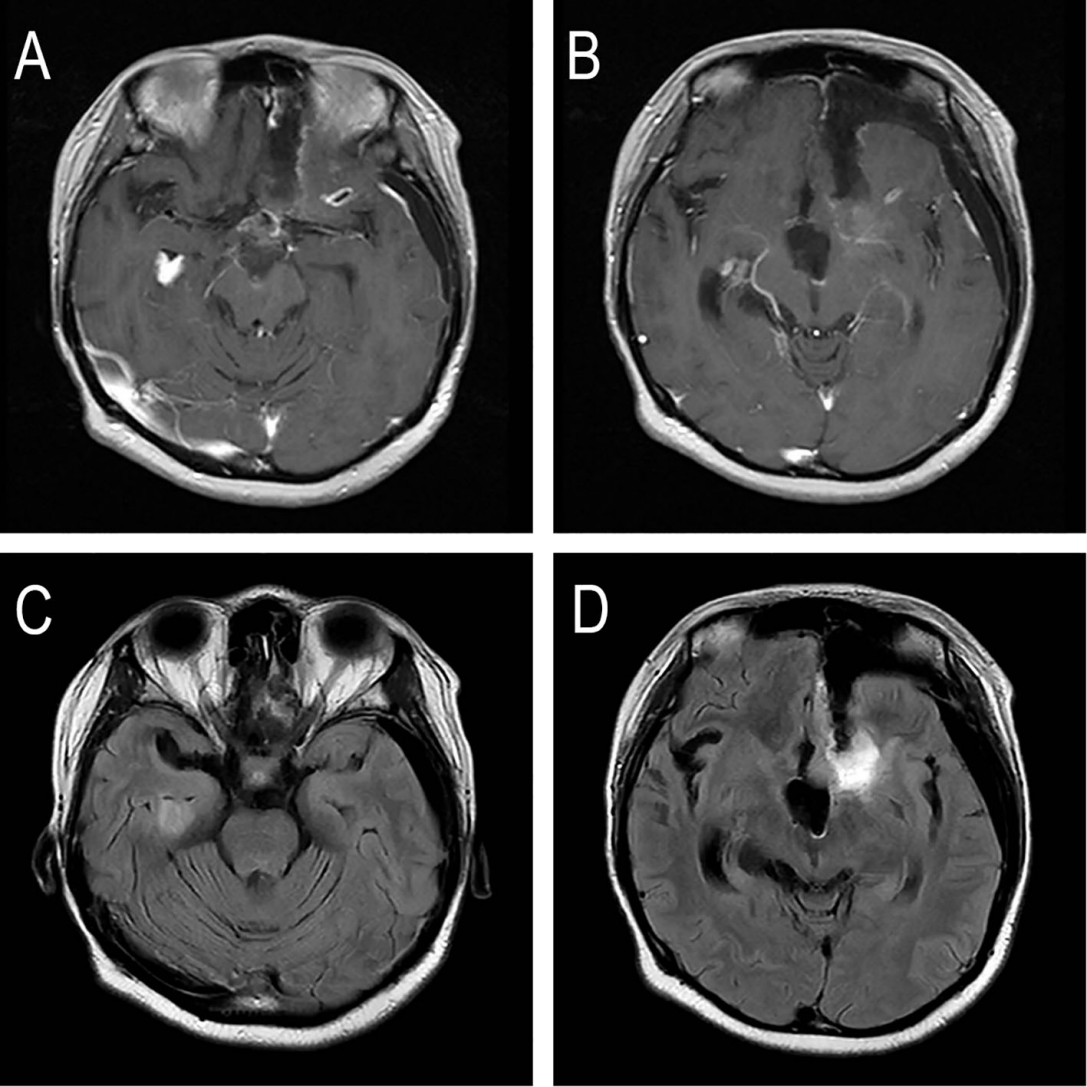
The patient’s MRI before re-radiotherapy. **(A, B)** T1-weighted contrast-enhanced MRI showing linear enhancement in the surgical cavity and the inferior horn of the right lateral ventricle. **(C, D)** T2-FLAIR MRI showing linear enhancement in the surgical cavity and the inferior horn of the right lateral ventricle.

Following the surgery, the patient underwent radiotherapy simulation positioning at the Department of Radiation Oncology, Shenzhen People’s Hospital, on August 21, 2025. On August 22, 2025, a pre-radiotherapy magnetic resonance imaging (MRI) examination was performed. The images demonstrated postoperative changes in the left frontal lobe, accompanied by mild linear enhancement around the resection cavity. The presence of a small ring-like enhancement focus within the left frontal lobe and a nodular enhancement focus in the inferior horn of the right lateral ventricle raises concern for residual tumor or cerebrospinal fluid metastasis; however, definitive assessment was limited by the unavailability of prior images for comparison. The study also confirmed significant communicating enlargement of both lateral ventricles, with a communication pathway identified between the left lateral ventricle, the surgical cavity, and the left frontotemporal subdural space. Expected postoperative changes of the left frontal bone were noted (**Figure 2**).

During simulation, the patient’s cognitive function was assessed using the European Organization for Research and Treatment of Cancer (EORTC) Quality of Life Questionnaire-Brain Neoplasm module (QLQ-BN20), which yielded a score of 37, and the EORTC Core Quality of Life Questionnaire (QLQ-C30 version 3.0), which yielded a score of 55. Physical examination upon admission showed the patient was conscious with a Karnofsky Performance Status (KPS) score of 60. The neurological examination revealed muscle strength of grade 3 in the lower limbs and grade 4 in the upper limbs, with normal muscle tone in all four limbs. No other relevant neurological symptoms or abnormalities in other organ systems were detected.

Concurrent chemoradiotherapy (CCRT) was initiated on September 3, 2025. Radiotherapy target volume delineation is as follows: The gross tumor volume 1 (GTV1) included the postoperative tumor cavity and the residual enhancing lesion in the left frontal lobe visible on T1-weighted contrast-enhanced images. The gross tumor volume 2 (GTV2) included the nodular enhancing lesion in the inferior horn of the right lateral ventricle. The clinical target volume 1 (CTV1) was created by expanding the GTV1 three-dimensionally by 1.5 cm, and the planning target volume 1 (PTV1) was generated by adding a 3 mm margin to the CTV1. The planning target volume 2 (PTV2) was created by expanding the GTV2 by 3 mm. The prescribed doses were 50 Gy in 25 fractions of 2 Gy to 95% of the PTV1 using a volumetric modulated arc therapy technique (VMAT), and 25 Gy in 5 fractions of 5 Gy to 99% of the PTV2 using stereotactic radiation therapy (SRT). The patient received concurrent chemotherapy consisting of daily oral temozolomide at a dose of 75 mg/m^2^. Additionally, recombinant human endostatin was administered via continuous infusion pump over 72 hours to mitigate radiotherapy-induced brain edema. The patient successfully completed a course of 25 fractions of adjuvant radiotherapy and was discharged following clinical improvement. Following discharge, adjuvant temozolomide chemotherapy was continued. Surveillance magnetic resonance imaging (MRI) is scheduled at three-month intervals. Treatment strategies will be adjusted based on disease evolution, and our team will maintain close follow-up. At the time of discharge, the Karnofsky Performance Status (KPS) score remained 60. Physical examination revealed muscle strength of grade 4 in all four limbs, normal muscle tone, and no other neurological symptoms or systemic abnormalities were detected.

**Figure 3:**
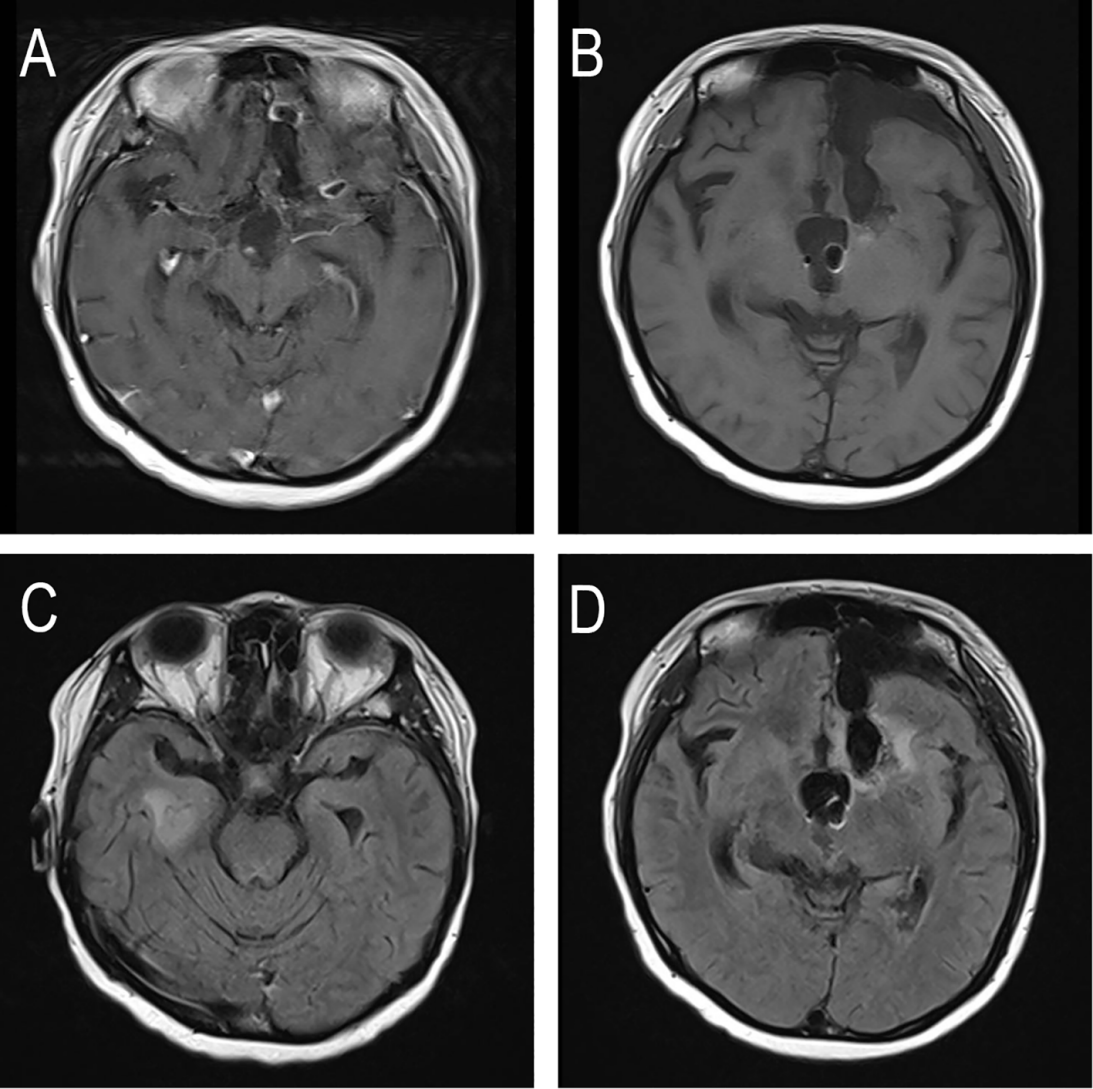
The patient’s MRI after re-radiotherapy. **(A, B)** T1-weighted contrast-enhanced MRI showing linear enhancement at the surgical margin and in the inferior horn of the right lateral ventricle. **(C, D)** T2-FLAIR MRI showing linear enhancement at the surgical margin and in the inferior horn of the right lateral ventricle.

On November 7, 2025, the patient returned for a follow-up examination. Compared to her discharge status, her mental state had improved, and her Karnofsky Performance Status (KPS) score remained stable at 60. No significant changes in cognitive function were noted on assessment. Follow-up MRI revealed a decrease in the linear enhancement within the operative area of the left frontal lobe. However, the previously identified small ring-like enhancement in the left frontal lobe and the nodular enhancement in the inferior horn of the right lateral ventricle were largely unchanged. The study also confirmed significant enlargement of both lateral ventricles, with communication between the left lateral ventricle, the surgical cavity, and the left frontotemporal subdural space, alongside expected postoperative changes of the left frontal bone (**Figure 3**). Given the stable imaging findings and clinical condition, continued periodic follow-up was recommended, with consideration for clinical trial enrollment upon any future disease progression.

## Discussion

Glioneuronal tumor with ATRX alteration, kinase fusion and anaplastic features (GTAKA) is a novel molecular subtype of glioma first defined in 2023. With only approximately 24 cases reported globally (1, 3, 4), it predominantly occurs in young patients (median age 19 years) and is most frequently located in the supratentorial region. Histopathologically, GTAKA is characterized by an “oligodendrocyte-like” cytomorphology within a glial background, alongside high-grade features such as frequent mitotic figures, microvascular proliferation, and focal necrosis. Immunohistochemically, the tumor cells typically express neuronal markers (SYN, MAP2) and glial markers (GFAP, Olig-2), with a notable loss of ATRX expression observed in approximately 85% of cases. Clinically, GTAKA exhibits aggressive behavior, with a reported median overall survival of 30.5 months (1). Despite this generally poor prognosis, a documented case demonstrated a remarkable response to an NTRK inhibitor, highlighting a promising therapeutic avenue for patients with this fusion (3). The extreme rarity of GTAKA means clinical data remain scarce, underscoring the need for further research to optimize diagnostic and therapeutic strategies for this emerging entity.

This case exhibits four notable characteristics. First, the patient developed two distinct pathological types of central nervous system tumors, suggesting underlying heterogeneity of cancer stem cells. Second, these two tumors occurred in different anatomical locations—the third ventricle and the left frontal lobe—indicating spatial heterogeneity. Third, the recurrent GTAKA tumor demonstrated a complex molecular genetic background, including an ATRX-inactivating mutation, dual NTRK fusions (MYO5A-NTRK3 and UBE2Q2-NTRK3), and a negative 1p/19q co-deletion status. Collectively, these findings provide a valuable research basis for interpreting the distinct biological behavior of GTAKA.

Although no standard therapeutic protocol exists for GTAKA, the treatment strategy for this case was based on the following evidence-based considerations. First, given that the pathology indicated this entity is equivalent to a WHO grade 3 tumor, adjuvant radiotherapy was administered to the patient with reference to current treatment strategies for high-grade neuroepithelial tumors. During radiotherapy, recombinant human endostatin was administered over two cycles to mitigate radiation-induced brain edema, as this agent has been shown to inhibit VEGF expression and reduce cerebral edema (5). Second, genomic testing revealed MGMT promoter methylation at 33.75%. Based on the treatment model for glioblastoma and the treatment experience of similar cases at Sun Yat-sen University Cancer Center, it was decided to combine the use of temozolomide for concurrent chemotherapy during radiotherapy and as adjuvant chemotherapy (6, 7). Furthermore, the patient’s NGS test indicated a NTRK gene fusion. Perhaps larotrectinib or entrectinib would be a good option. However, due to the patient’s financial situation and the accessibility of the drugs, we have not used these medications at present. If the patient’s tumor recurs in the future, we will consider using them after a further comprehensive assessment (8).

The patient had a history of radiotherapy following a previous astrocytoma resection, making the current radiotherapy a case of re-irradiation. Radiation dose accumulates in organs at risk (OARs) over successive courses, and while this cumulative effect can attenuate over time, precise control over the dose to OARs is critical in re-irradiation. Typically, excessive cumulative dose can lead to reversible or irreversible damage to these OARs (9). Previous studies suggest that the detrimental effect of the initial radiotherapy dose on tolerance may become insignificant when the interval before re-irradiation exceeds three years, leading to the proposal of a dose discounting rate for OARs in re-irradiation scenarios (10). Consequently, we applied these principles to constrain the dose to the OARs in the present case. We believe that a regimen of 50 Gy delivered in standard fractions strikes a balance between therapeutic efficacy and the risk of neurotoxicity.

This case has a major limitation: the original pathological specimen from 2003 was unavailable for retrieval and re-evaluation. Consequently, we cannot entirely rule out the possibility that the current GTAKA originated from an earlier misdiagnosed tumor. However, considering the occurrence of the two tumors in distinct anatomical locations (the third ventricle *vs*. the left frontal lobe) and the 22-year disease-free interval, our findings more strongly support the interpretation that this represents either a second primary tumor arising from heterogeneous cancer stem cells or an exceptionally late true recurrence. Although the possibility of an initial misdiagnosis cannot be excluded, the treatment administered for the current diagnosis of GTAKA was considered effective in the short term.

Currently, there is no designated WHO classification for this specific pathological subtype of central nervous system tumor. The present case represents the first reported instance of GTAKA in China. The management of this disease is still in an exploratory phase, and further clinical data collection and dedicated clinical trials are essential to advance our understanding and develop effective treatment strategies.

## Conclusion

We report a unique case of a glioneuronal tumor with anaplastic features and kinase fusion (GTAKA) that emerged as a new lesion 22 years after the initial treatment for a low-grade glioma. This case underscores the critical role of integrating histopathological and molecular profiling in diagnosing complex central nervous system tumors. Comprehensive molecular analysis unequivocally established the diagnosis, revealing a distinctive genetic background characterized by dual NTRK3 fusions and an ATRX mutation. Furthermore, this report demonstrates that a meticulously planned regimen of postoperative re-irradiation concurrent with temozolomide chemotherapy is a feasible treatment strategy that led to short-term disease stabilization with preserved performance status. Finally, it is suggested that the treatment of GTAKA requires personalized approaches. Moreover, the potential of NTRK inhibitors as future treatment options merits further exploration in clinical trials. This report provides valuable real-world evidence for this emerging tumor subtype, which is growing but still limited in the literature.

## Data Availability

The datasets presented in this article are not readily available because no. Requests to access the datasets should be directed to MS, sunmengqi1990@126.com.
